# I-CPA: An Improved Carnivorous Plant Algorithm for Solar Photovoltaic Parameter Identification Problem

**DOI:** 10.3390/biomimetics8080569

**Published:** 2023-11-27

**Authors:** Ayşe Beşkirli, İdiris Dağ

**Affiliations:** 1 Department of Computer Engineering, Eskişehir Osmangazi University, 26000 Eskişehir, Türkiye; 2 Department of Computer Engineering, Karamanoğlu Mehmetbey University, 70200 Karaman, Türkiye

**Keywords:** parameter extraction, parameter identification, photovoltaic models, solar cells, solar module, carnivorous plant algorithm

## Abstract

The carnivorous plant algorithm (CPA), which was recently proposed for solving optimization problems, is a population-based optimization algorithm inspired by plants. In this study, the exploitation phase of the CPA was improved with the teaching factor strategy in order to achieve a balance between the exploration and exploitation capabilities of CPA, minimize getting stuck in local minima, and produce more stable results. The improved CPA is called the I-CPA. To test the performance of the proposed I-CPA, it was applied to CEC2017 functions. In addition, the proposed I-CPA was applied to the problem of identifying the optimum parameter values of various solar photovoltaic modules, which is one of the real-world optimization problems. According to the experimental results, the best value of the root mean square error (RMSE) ratio between the standard data and simulation data was obtained with the I-CPA method. The Friedman mean rank statistical analyses were also performed for both problems. As a result of the analyses, it was observed that the I-CPA produced statistically significant results compared to some classical and modern metaheuristics. Thus, it can be said that the proposed I-CPA achieves successful and competitive results in identifying the parameters of solar photovoltaic modules.

## 1. Introduction

**Figure 1:**
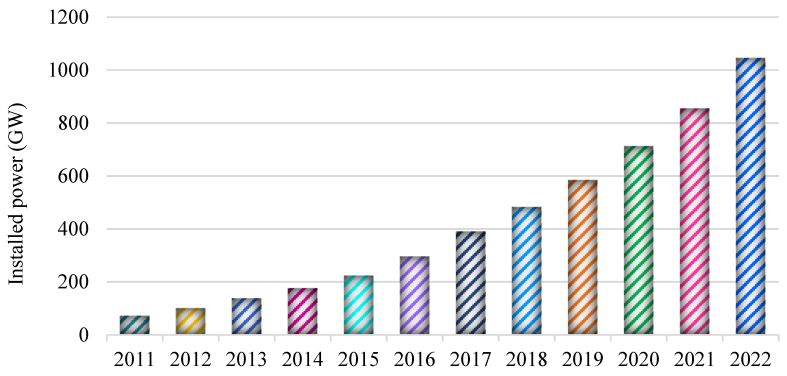
Installed power capacity of solar panels in the world according to years.

Energy, which manifests itself in all areas of human life, is becoming increasingly important today [1]. The world’s increasing population, developing industry, and advances in technology are increasing the need for energy in developed and developing countries [2]. Considering the increasing energy need, energy crises, and environmental problems in the world, the importance of renewable energy sources such as wind, solar, geothermal, and wave, which are reliable, inexhaustible, and clean, is increasing [3]. Among these sources, solar energy is one of the renewable energy sources that attracts a lot of attention and contributes significantly to energy production [4]. According to the report of the International Renewable Energy Agency, it is seen in Figure 1 that the installed power capacity of solar panels for obtaining electrical energy from the sun is gradually increasing across the world [5]. 

**Figure 2:**
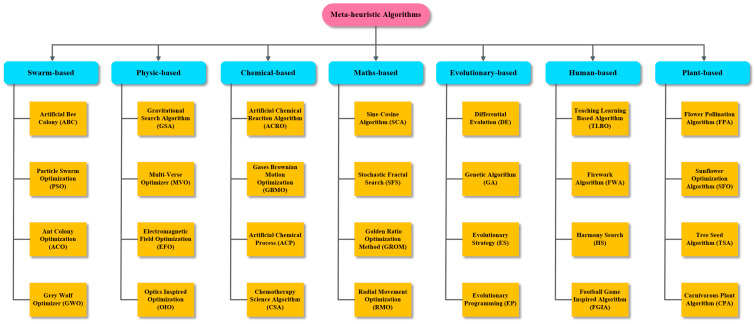
Some metaheuristic algorithms in the literature and their classification.

Since there is a worldwide trend towards solar energy, interest in solar panels has increased and scientists have been in search of how to increase the efficiency obtained from solar panels [6]. Solar panels are composed of photovoltaic cells, which enable the generation of electrical energy from sunlight [7]. The performance evaluation, simulation, and optimization of photovoltaic models also depends on the identification of the optimum parameters of photovoltaic models [8]. There are three different photovoltaic models commonly used in the literature [9]: single diode, double diode, and PV module models. Many metaheuristic algorithms have been used in the literature to identify the optimum parameter values of these models. Some of the metaheuristic algorithms in the literature are given in Figure 2.

The nature-inspired metaheuristic algorithms in Figure 2 are presented in seven main categories in this study. The first category consists of swarm-based algorithms, inspired by the social behavior of swarming communities. Some of these algorithms are the artificial bee colony (ABC) [10], grey wolf optimizer (GWO) [11], ant colony optimization (ACO) [12], and particle swarm optimization (PSO) [13]. The second category consists of physics-based algorithms inspired by the laws of physics. Some of them are the multi-verse optimizer (MVO) [14], gravitational search algorithm (GSA) [15], electromagnetic field optimization (EFO) [16], and optics inspired optimization (OIO) [17]. The third category includes chemistry-based algorithms that are inspired by the laws of chemistry. Some of them are the artificial chemical reaction algorithm (ACRO) [18], gases brownian motion optimization (GBMO) [19], artificial chemical process (ACP) [20], and chemotherapy science algorithm (CSA) [21]. The fourth category includes math’s-based algorithms, which are inspired by mathematical rules. Some of these algorithms are the sine-cosine algorithm (SCA) [22], stochastic fractal search (SFS) [23], golden ratio optimization method (GROM) [24], and radial movement optimization (RMO) [25]. The fifth category includes evolutionary-based algorithms inspired by biological phenomena in nature. Some of them are the differential evolution (DE) [26], genetic algorithm (GA) [27], evolutionary strategy (ES) [28], and evolutionary programming (EP) [29]. The sixth category consists of human based algorithms inspired by human behavior. Some of these algorithms are the teaching learning based algorithm (TLBO) [30], firework algorithm (FWA) [31], harmony search (HS) [32], and football game inspired algorithm (FGIA) [33]. The last category includes plant-based algorithms, which are inspired by the behavior of plants in nature. Some of them are the flower pollination algorithm (FPA) [34], sunflower optimization algorithm (SFO) [35], tree seed algorithm (TSA) [36], and carnivorous plant algorithm (CPA) [37].

Many metaheuristic algorithms have been proposed in the literature for solving optimization problems. However, the basic forms of algorithms are sometimes insufficient for solving optimization problems [38,39]. In addition, algorithms may tend to get stuck in local minima in identifying the parameters of PV module models [40,41]. Therefore, in order to improve the performance of the CPA, the local search capability of the CPA has been improved. Thus, we aimed to minimize the tendency of the CPA to get stuck in local minima. The proposed method, with the improvement made in the CPA, is called the I-CPA. The effectiveness and performance of the I-CPA are tested on the parameter optimization of four different solar photovoltaic panels and compared with the results obtained by the basic CPA. The obtained results were run for 30 runtimes until the maximum number of function evaluations (MaxFEs) termination condition was met. The convergence curves, box plots, I-V characteristics, and P-V characteristics of the obtained results are presented in the related sections. Furthermore, the performance of the I-CPA is compared with the results of some classical and modern metaheuristic algorithms, such as the differential evolution algorithm (DE) [26], particle swarm optimization (PSO) [13], carnivorous plant algorithm (CPA) [37], coati optimization algorithm (COA) [42], and skill optimization algorithm (SOA) [43]. The main contributions of this study are as follows:

- A teaching factor (TF) strategy has been added to the CPA in order to minimize getting stuck in local minima and produce more stable results. Thus, an improved CPA (I-CPA) is proposed, aiming to introduce it to the literature.
- The performance and success of the proposed I-CPA are first tested on CEC2017 functions. Then, the proposed I-CPA is applied to identify the parameters of solar photovoltaic modules.
- The performance of the I-CPA is compared not only with the basic CPA but also with the results of some classical and modern metaheuristics. The comparison results are supported by convergence and box plots.
- The Friedman mean rank test was performed to show the ranking of the I-CPA among the compared algorithms and the significance of the results.
- Experimental results and statistical analyses show that the proposed I-CPA is an effective and competitive method.

This paper is organized as follows: Section 2 presents the literature studies on both the basic CPA and photovoltaic models. Section 3 describes the basic CPA. In Section 4, the added strategy to improve the performance of the CPA is described. Section 5 presents the PV models used in the study, the mathematical equations of these models, and the objective function of the problem. In Section 6, the performance comparison of the proposed I-CPA and other algorithms on CEC2017 functions is presented. Section 7 presents detailed analyses of the experimental results. In Section 8, the results of the study are interpreted and suggestions for future studies are given.

## 2. Related Works

In the literature, there are many studies on parameter extraction of photovoltaic models with metaheuristic algorithms. Boğar [44] integrated the least squares method into the chaos game optimization algorithm and proposed a new hybrid algorithm called the CGO-LS. To verify the effectiveness of the CGO-LS, it was applied to the parameter estimation problem of PV models. The performance of the CGO-LS was compared with both the basic CGO and the results reported in the literature. According to the comparison result, it is stated that the CGO-LS is a competitive method. Ali et al. [45] proposed an atomic orbital search algorithm to extract unknown parameters of various solar cells. The proposed algorithm was applied to two different solar cells and performance analyses were performed based on RMSE values. In their comprehensive analyses, they explained that the proposed algorithm obtained the best result compared to the other algorithms in the extraction of unknown parameters of solar cells. Duan et al. [46] carried out a study aiming at parameter extraction of the photovoltaic model using the nutcracker optimizer algorithm. They used three different photovoltaic models for this purpose. To assess the performance of the proposed algorithm on these models, they compared it with three popular algorithms in the literature, such as the whale optimization algorithm, fireworks algorithm, and particle swarm optimization. According to the experimental results, the least error value was obtained with the proposed algorithm in all models and, thus, the efficiency in parameter extraction of photovoltaic models is increased by the suggested algorithms. El-Mageeda et al. [41] introduced a new method called IQSODE by adapting an improved queuing search optimization method to the differential evolution algorithm. This proposed method was used to extract the parameters of models such as single diode, double diode, and PV module. They compared the results of this method with those of other algorithms in the literature, stating its superior performance both statistically and in terms of convergence speed. It was concluded that the proposed method is a suitable alternative for parameter extraction of photovoltaic models. Vais et al. [47] employed the dandelion optimization algorithm (DOA), a bio-inspired algorithm, to analyze the parameters of two different models: single diode and double diode, of various panel types. The results obtained with the proposed method were compared with those of both the analytical methods and also other algorithms in the literature. The comparisons indicated that the proposed method is a successful parameter estimation tool, exhibiting a sufficient performance compared to other algorithms. El-Dabah et al. [4] utilized northern goshawk optimization to identify nine unknown parameters of a three-diode model of three commercial modules. The results obtained by the proposed method were compared with the results of some algorithms in the literature. According to the comparison results, the proposed method is said to be competitive compared to other algorithms in terms of convergence speed and accuracy. In the work presented by Dkhichi [48], five parameters of the photovoltaic system are extracted by hybridizing the Levenberg–Marquardt and simulated annealing algorithm. When the results of the proposed method were compared with the results of newly proposed algorithms in the literature, it was stated that the suggested method proved its superiority in terms of accuracy and robustness. Chaib et al. [49] estimated the parameters of various photovoltaic models using the Harris hawks algorithm, which has the advantages of good search ability, high convergence speed, and high efficiency compared to classical methods. Estimation procedures were carried out according to various weather conditions. According to the experimental results, the lowest error value was obtained with the HHO, thus proving its superiority in solving this problem. Maden et al. [50] conducted a study on the estimation of parameters of photovoltaic cells. In this study, the values of single diode and double diode parameters were obtained by using the squirrel search algorithm (SSA). According to the results obtained, it is stated that SSA provides superior success compared to its competitors in the literature and increases the efficiency of photovoltaic systems. Sharma et al. [51] used metaheuristic algorithms to infer the parameters of four different panel types. According to the experimental results, they realized that the algorithms that provide success in different types of panel change. For this reason, they recommended further improvement of the algorithms. Qaraad et al. [52] utilized the moth flame algorithm (MFO) for inferring the parameters of photovoltaic systems. In addition, the IMFOL algorithm was proposed by adding the local escape operator (LEO) mechanism to increase both the population diversity and the exploration capability of the MFO algorithm. The results obtained by the proposed methods were compared with the results of algorithms in the literature. According to the comparison, the IMFOL algorithm is better in parameter extraction of PV systems. Thus, the IMFOL algorithm is said to be an effective alternative method, exhibiting prosperity in speed, stability, and accuracy.

In the literature, many studies deal with solving optimization problems using the CPA. Wang et al. [53] proposed a new method called the CPA-HDM by adding various improvement strategies to the exploration phase, which is the growth phase, to balance exploration and exploitation capabilities in the CPA. They assessed the performance of this proposed method on the travelling salesman problem. According to the experimental results and statistical analyses, the CPA-HDM method has superior performance. Wang et al. [54] improved the CPA with the Levy mutation and similarity-removal operation in order to solve problems such as the CPA getting stuck in local minima; the solutions obtained were a little poor. Thus, it was stated that they increased the convergence speed of the CPA and reduced the sticking to local minima. In order to prove this, they tested their proposed method on test functions and engineering problems. According to the experimental results, it is said that the solution quality of the proposed method was improved. In the work presented by Yang and Zhang [55], the CPA was transformed into a multi-objective algorithm in order to solve multiple objectives simultaneously. They also improved the exploitation phase of the multi-objective CPA to avoid local minima and increase the convergence speed. The performance of the proposed algorithm was tested on various test functions and the multi-objective CPA is said to be a competitive method. Wang et al. [56] optimized the artificial neural network with CPA using four input data. From this, they predicted the bond strength of wood and also predicted the surface roughness of wood. In the experiments, both the neural network and the neural network optimized with CPA were used. According to the experimental results, the results obtained from the CPA-optimized neural network are said to be better. Zhang et al. [57] transformed the CPA into a discrete structure to solve the travelling salesman problem. They made various improvements to the discrete CPA to obtain better results. As a result of experimental analyses, the proposed method is said to produce significantly better results. Peng et al. [58] added multiple strategies to CPA in order to overcome some of its shortcomings and improve its performance. The improved algorithm was tested on some benchmark functions and its success was verified. In addition, engineering problems were solved with their proposed method, and they stated that the results obtained were successful. Thus, it is stated that the proposed method is an alternative for solving competitive and optimization problems. Yang et al. [59] improved on the existing formulation for the estimation of carbon emission and developed a combined estimation model. Carbon emissions were estimated using the CPA in this model and it was stated that the results obtained were superior.

## 3. Carnivorous Plant Algorithm

The carnivorous plant algorithm (CPA) is a new bio-inspired metaheuristic algorithm that simulates the survival process of carnivorous plants proposed by Ong [37] in 2021. The CPA focuses on the idea of how carnivorous plants adapt to survive in harsh environmental conditions. In the CPA, there are basically four parts: the initialization phase, classification and grouping phase, growth phase, and reproduction phase. The main stages of the CPA are growth (exploration) and reproduction (exploitation).

### 3.1. Initialization Phase

The CPA is initialized by randomly distributing a population of *N* individuals consisting of carnivorous plants (*nCPlant*) and prey (*nPrey*). The matrix of the positions of individuals in the initial population is given in Equation (1).

(1) $$X=\left[\begin{array}{ccc} X_{1,1} & X_{1,2}\;\;\;\;\cdots & X_{1,D}\\ X_{2,1} & X_{2,2}\;\;\;\;\cdots & X_{2,D}\\ \vdots & \;\;\vdots \;\;\;\;\;\;\;\;\;\ddots & \vdots \\ X_{N,1} & X_{N,2}\;\;\;\cdots & X_{N,D} \end{array}\right]$$

where, the number of dimensions is expressed by *D* and the sum of *nCPlant* and *nPrey* is denoted by *N*. Thus, the initial population is randomly initialized using Equation (2).

(2) $$Individual_{i,j}=Lb_{j}+\left(Ub_{j}-Lb_{j}\right)\times rand$$

where *i* = 1, 2, …, *N* and *j* = 1, 2, …, *D*. The lower bound and upper bound of the search space are represented by *Lb* and *Ub*, respectively. *rand* is a randomly generated number in the range [0, 1].

### 3.2. Classification and Grouping Phase

In this section, after the individuals are sorted in ascending order according to the fitness value, the *nCPlant* solutions at the top of the population are expressed as carnivorous plants (*CP*), while the remaining solutions are expressed as *nPrey*. The *nPrey* number is *k* times the *nCPlant* in the population and is an integer number. *k* is given in Equation (3).

(3) $$k=\frac{nPrey}{nCPlant}$$

where *nCPlant* is also the number of groups. The number of preys per group is denoted by *k*. The sum of *nCPlant* and *nPrey* is the population size (*N*). To illustrate the grouping process with an example [54], when the number of *nCPlants* is 2 and the number of *nPreys* is 8, the population size is 10. The number of preys per group is 4.

### 3.3. Growth Phase

This part consists of the exploration phase. In order to obtain the nutrients they need, carnivorous plants first release a pleasant smell and try to trap their prey. Then, the carnivorous plant takes the necessary nutrients for its growth by digesting the trapped prey. However, some prey may escape from the trap. In order to control this situation, an attraction rate (*ar*) parameter is included in the algorithm. If the value of the *ar* parameter is greater than a randomly selected number between 0 and 1, Equation (4) is used and if it is smaller, Equation (5) is used.

(4) $$NewCP_{i,j}=growth\times CP_{i,j}+\left(1-growth\right)\times Prey_{v,j}\;growth=growth\text{\_}rate\times rand_{i,j}$$

(5) $$NewPrey_{i,j}=growth\times Prey_{u,j}+\left(1-growth\right)\times Prey_{v,j},u\neq v\;growth=\{\begin{array}{cc} growth\text{\_}rate\times rand_{i,j} & f\left(prey_{v}\right)>f\left(prey_{u}\right)\\ 1-growth\text{\_}rate\times rand_{i,j} & f\left(prey_{v}\right)<f\left(prey_{u}\right) \end{array}$$

where $CP_{i,j}$ is the current carnivorous plant and $Prey_{v,j}$ is the randomly selected individual. $Prey_{u,j}$ refers to another randomly selected individual from group *i.*

### 3.4. Reproduction Phase

This part consists of the exploitation stage. Carnivorous plants use the nutrients they obtain for growth and reproduction. The best individual in the population, the first ranked individual, is allowed to reproduce. With this process, only the best solution is focused on in the CPA. The mathematical formula of the reproduction process of the first ranked individual is given in Equation (6).

(6) $$NewCP_{i,j}=CP_{1,j}+Reproduction\text{\_}rate\times rand_{i,j}\times mate_{i,j}\;mate_{i,j}=\{\begin{array}{cc} CP_{v,j}-CP_{i,j} & f\left(CP_{i}\right)>f\left(CP_{v}\right)\\ CP_{i,j}-CP_{v,j} & f\left(CP_{i}\right)<f\left(CP_{v}\right) \end{array},i\neq v\neq 1$$

where $CP_{1,j}$ is the best solution and $CP_{v,j}$ is the randomly selected carnivorous plant. The process in this step is repeated for *nCPlant* values. The pseudo code of CPA is given in Algorithm 1 [37,57].
**Algorithm 1:** Pseudo code of CPA.**Input:**   The population size N     The population size of carnivorous plants: nCPlant     The population size of prey: nPrey     Group_iter: gi     Attraction_rate: ar     Growth_rate: gr     Reproduction_rate: rr     Maximum iteration: Maxiter **1.** Generate initial individuals in the population **2.** Calculate the fitness value and sort based on the fitness value **3.** Identify the best individual, g* as the first rank carnivorous plant(CP)  **WHILE** iter < Maxiter **4.** Set top nCPlant individuals as carnivorous plants The remaining nPrey individuals as prey Group the carnivorous plants and prey /*Growth process  **FOR** i = 1:nCPlant   **FOR** Group_cycle = 1:gi    **IF** ar > a generated random number     Generate new carnivorous plant using Equation (4)    **ELSE**     Generate new prey using Equation (5)   **END FOR**  **END FOR** /*Reproduction process  **FOR** i = 1:nCPlant   Generate new carnivorous plant based on the first rank CP using Equation (6)  **END FOR** **5.** Evaluate the fitness of each new CP and new prey **6.** Combine the previous and newly generated CPs and preys **7.** Sort the individuals and select top n-ranked individuals to next generation **8.** Identify the current best individual, g* as the first rank carnivorous plant  **END WHILE** **Output:**   The best solution and g*

## 4. Improved Carnivorous Plant Algorithm

**Figure 3:**
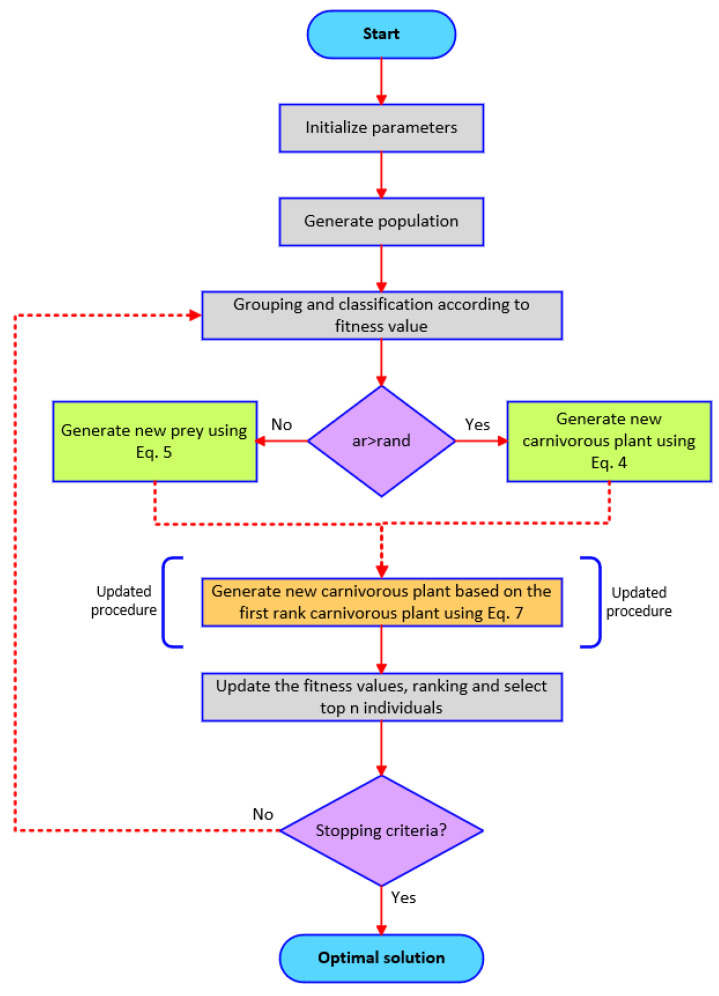
Flowchart of the I-CPA.

The performance of metaheuristic algorithms depends on the ability to search efficiently in the search space. There are two search capabilities in the algorithms: local search and global search. These are called exploitation and exploration, respectively. While global search explores all regions in the search space, local search is responsible for obtaining a better result by searching around the global best result [60]. The performance of the algorithm increases by balancing both global search and local search [61]. As the complexity of a problem increases, it becomes more difficult to reach the best result. For this reason, some improvements are made on the basic algorithm in order for the algorithm to produce more effective results. In order to provide a better balance between the exploration and exploitation capabilities of the CPA method, an effective improvement is made by adding the teaching factor (*T_F_*) strategy [30,62] in the exploitation phase of the CPA. In this way, the local search performance of the CPA is increased by searching for better solutions around the best result obtained by the global search. Thus, both the exploration and exploitation capabilities of the CPA are more balanced, contributing to a more stable and a better result of the CPA. The improvement in the local search equation is given in Equation (7).

(7) $$NewCP_{i,j}=CP_{1,j}+Reproduction\text{\_}rate\times rand_{i,j}\times mate_{i,j}\;mate_{i,j}=\{\begin{array}{cc} CP_{v,j}-T_{F}*CP_{i,j} & f\left(CP_{i}\right)>f\left(CP_{v}\right)\\ CP_{i,j}-CP_{v,j} & f\left(CP_{i}\right)<f\left(CP_{v}\right) \end{array},i\neq v\neq 1\;T_{F}=round(1+rand())$$

where the strategy $T_{F}$ was added to the $CP_{i,j}$ in the $mate_{i,j}$ equation to improve the exploitation capability of the CPA. The strategy $T_{F}$ is an integer, and the number 1 or 2 is chosen randomly. The $T_{F}$ strategy is an efficient method and has been used in many studies in the literature [42,63,64,65,66,67]. This method contributes to the balance between exploration and exploitation and allows for the algorithm to achieve successful results. The pseudo code of the improved CPA (I-CPA) is given in Figure 3.

## 5. Photovoltaic Models and Objective Functions

In this section, information about the single diode (SD) model, double diode (DD) model, and PV module (PVM) model of solar cells are given. In addition, equivalent circuits, mathematical equations, and objective functions of these models are presented under the relevant titles [7].

### 5.1. Single Diode (SD) Model

The single diode model, which is one of the most widely used solar cell models, consists of five parameters ($I_{ph},I_{sd},R_{s},R_{sh},n$). The output current of this model is calculated according to Kirchhoff’s Current Law, as in Equation (8). The diode current ($I_{d}$) and shunt resistor current ($I_{sh}$) in this equation are calculated by the formula in Equation (9) and Equation (10), respectively. The extended formula of the output current is given in Equation (11) and the objective function of this model is given in Equation (12).

(8) $$I_{L}=I_{ph}-I_{d}-I_{sh}$$

(9) $$I_{d}=I_{sd}\times \left[\exp\left(\frac{q\times \left(V_{L}+R_{s}\times I_{L}\right)}{n\times k\times T}\right)-1\right]$$

(10) $$I_{sh}=\frac{V_{L}+R_{s}\times I_{L}}{R_{sh}}$$

(11) $$I_{L}=I_{ph}-I_{sd}\times \left[\exp\left(\frac{q\times \left(V_{L}+R_{s}\times I_{L}\right)}{n\times k\times T}\right)-1\right]-\frac{V_{L}+R_{s}\times I_{L}}{R_{sh}}$$

(12) $$\{\begin{array}{c} \;f_{i}\left(V_{L},I_{L},X_{SD}\right)=I_{ph}-I_{sd}\times \left[exp\left(\frac{q\times \left(V_{L}+R_{s}\times I_{L}\right)}{n\times k\times T}\right)-1\right]-\frac{V_{L}+R_{s}\times I_{L}}{R_{sh}}-I_{L}\\ X_{SD}=\left\{I_{ph},I_{sd},R_{s},R_{sh},n\right\} \end{array}$$

**Figure 4:**
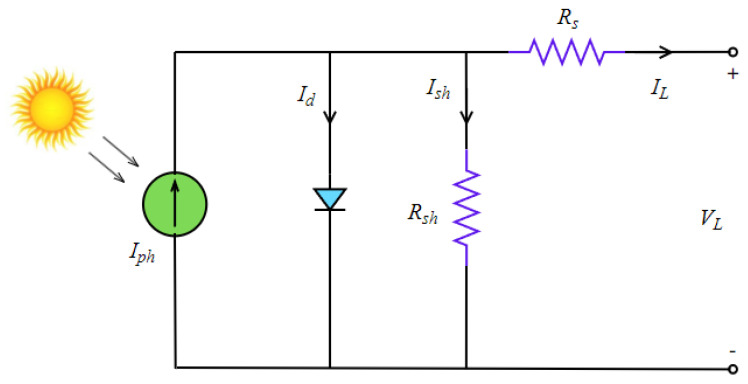
Equivalent circuit of the SD model.

The parameter n is the ideality factor, $R_{sh}$ is the shunt resistance, $R_{s}$ is the series resistance, $I_{sd}$ is the saturation current, and $I_{ph}$ is the photo generated current. The equivalent circuit of the model is given in Figure 4.

### 5.2. Double Diode (DD) Model

The double diode model considering recombination current loss consists of seven parameters ($I_{ph},I_{sd1},I_{sd2},R_{s},R_{sh},n_{1},n_{2}$). The output current of this model is calculated as in Equation (13). The diode current ($I_{d1}$ and $I_{d2}$) and shunt resistor current ($I_{sh}$) in this equation are calculated by the formula in Equations (14), (15) and (16), respectively. The extended formula of the output current is given in Equation (17) and the objective function of this model is given in Equation (18).

(13) $$I_{L}=I_{ph}-I_{d1}-I_{d2}-I_{sh}$$

(14) $$I_{d1}=I_{sd1}\times \left[\exp\left(\frac{q\times \left(V_{L}+R_{s}\times I_{L}\right)}{n_{1}\times k\times T}\right)-1\right]$$

(15) $$I_{d2}=I_{sd2}\times \left[\exp\left(\frac{q\times \left(V_{L}+R_{s}\times I_{L}\right)}{n_{2}\times k\times T}\right)-1\right]$$

(16) $$I_{sh}=\frac{V_{L}+R_{s}\times I_{L}}{R_{sh}}$$

(17) $$I_{L}=I_{ph}-I_{sd1}\times \left[\exp\left(\frac{q\times \left(V_{L}+R_{s}\times I_{L}\right)}{n_{1}\times k\times T}\right)-1\right]-I_{sd2}\times \left[\exp\left(\frac{q\times \left(V_{L}+R_{s}\times I_{L}\right)}{n_{2}\times k\times T}\right)-1\right]-\frac{V_{L}+R_{s}\times I_{L}}{R_{sh}}$$

(18) $$\{\begin{array}{c} f_{i}\left(V_{L},I_{L},X_{DD}\right)=I_{ph}-I_{sd1}\times \left[exp\left(\frac{q\times \left(V_{L}+R_{s}\times I_{L}\right)}{n_{1}\times k\times T}\right)-1\right]\\ -I_{sd2}\times \left[exp\left(\frac{q\times \left(V_{L}+R_{s}\times I_{L}\right)}{n_{2}\times k\times T}\right)-1\right]-\frac{V_{L}+R_{s}\times I_{L}}{R_{sh}}-I_{L}\\ X_{DD}=\left\{I_{ph},I_{sd1},I_{sd2},R_{s},R_{sh},n_{1},n_{2}\right\} \end{array}$$

**Figure 5:**
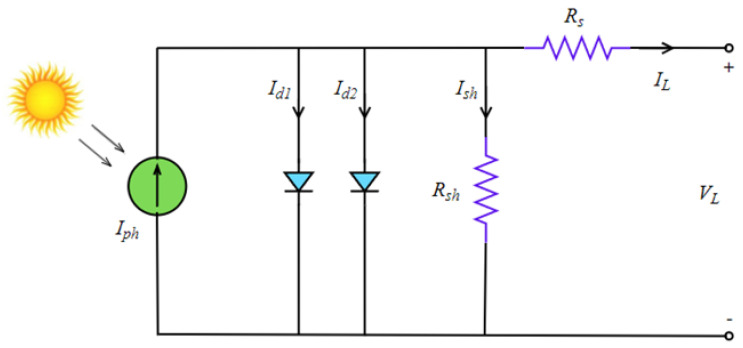
Equivalent circuit of the DD model.

In addition to the single diode model parameters, $I_{sd1}$, $I_{sd2}$, $n_{1}$, and $n_{2}$ parameters are included in this model. These parameters represent the diffusion current, saturation current, diode ideality factor, and recombination diode ideality factor, respectively. The equivalent circuit of the DD model is given in Figure 5.

### 5.3. PV Module (PVM) Model

The PVM model, which is based on the single diode model, consists of cells connected in series and parallel. The mathematical formula for the output current of the PV module is given in Equation (19) and the objective function of the model is given in Equation (20).

(19) $$I_{L}/N_{p}=I_{ph}-I_{sd}\times \left[\exp\left(\frac{q\times \left(V_{L}/N_{s}+R_{s}\times I_{L}/N_{p}\right)}{n\times k\times T}\right)-1\right]-\frac{V_{L}/N_{s}+R_{s}\times I_{L}/N_{p}}{R_{sh}}$$

(20) $$\{\begin{array}{c} f_{i}\left(V_{L},I_{L},X_{PVM}\right)=I_{ph}-I_{sd}\times \left[exp\left(\frac{q\times \left(\frac{V_{L}}{N_{s}}+\frac{R_{s}\times I_{L}}{N_{p}}\right)}{n\times k\times T}\right)-1\right]\\ -\frac{\frac{V_{L}}{N_{s}}+\frac{R_{s}\times I_{L}}{N_{p}}}{R_{sh}}-\frac{I_{L}}{N_{p}}\\ X_{PVM}=\left\{I_{ph},I_{sd},R_{s},R_{sh},n\right\} \end{array}$$

**Figure 6:**
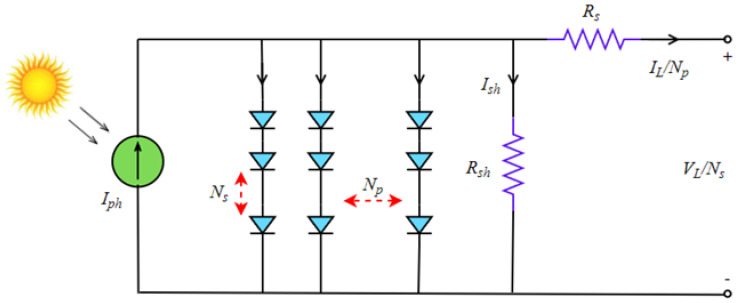
Equivalent circuit of PVM model.

The equivalent circuit of solar cells connected to each other as $N_{s}$ × $N_{p}$ is given in Figure 6. Where $N_{s}$ represents series connected cells, while $N_{p}$ represents parallel connected cells.

After determining the parameter values of the single diode, double diode and PV module models, the formula for the root mean square error (RMSE) of the optimization problem, which aims to minimize the error between simulation data and standard data, is given in Equation (21), where *N* is the number of standard data.

(21) $$\begin{array}{c} RMSE\left(X\right)=\sqrt{\frac{1}{N}\sum_{i=1}^{N}f_{i}{\left(V_{L},I_{L},X\right)}^{2}}\\ X=\left[X_{SD},\;X_{DD},\;X_{PVM}\right] \end{array}$$

## 6. Performance Comparison of the Proposed I-CPA and Other Algorithms on CEC2017

In this section, CEC2017 test functions are used to test the performance of the proposed I-CPA. Two of the CEC2017 test functions are unimodal, seven are multimodal, ten are hybrid, and the remaining ten are composition functions. CEC2017 test functions consisting of 29 functions in total are given in Table 1 [68].

All the algorithms used in this study were run 30 times under the same conditions. The mean and standard deviation values obtained as a result of the runs are given in Table 2. In addition, the results obtained by the proposed I-CPA are compared with the results obtained by the basic CPA, SOA, COA, PSO, and DE algorithms. Moreover, in order to show the significance of the obtained results, the results of all the algorithms are subjected to the Friedman mean rank test [69]. The statistical ranking results of the algorithms are given in Table 2.

The results in Table 2 show that the I-CPA obtained the best mean values in most of the CEC2017 test functions. According to the Friedman mean rank test, the I-CPA was the first algorithm to perform well in rank with a value of 1.31. Then, the PSO was the second-best performing algorithm with a value of 2.45. The third best performing algorithm was the DE algorithm with an FMR value of 3.03. According to the FMR test, the basic CPA ranked 5th among all algorithms with a value of 4.72. The p-value obtained as a result of the Friedman mean rank test is less than 0.05, which shows that the I-CPA produces statistically significant results in CEC2017 test functions compared to other algorithms.

## 7. Experimental Results

In this section, the proposed I-CPA and the basic CPA are applied to determine the optimum parameter values for SD, DD, and PV module models. For this purpose, four different solar photovoltaic module models popular in the literature were selected. The lower and upper bound (LB and UB) values of these models are given in Table 3. The *nCPlant* and *nPrey* values of the algorithms in the study were 2 and 8, respectively, and the sum of both parameters gives the number of populations, and its value was 10. MaxFEs was taken as 50,000. The study was run for 30 runtimes under equal conditions. The experimental results, convergence, and box plots obtained with the proposed I-CPA and the basic CPA according to these conditions are presented under the relevant titles.

Where $I_{sc}$ value in the table represents the short circuit current. The formula of $I_{sc}$ is given in Equation (22).

(22) $$I_{sc}\left(G,T\right)=I_{sc-STC}\frac{G}{G_{STC}}+\alpha \left(T-T_{STC}\right)$$

where G is the irradiance level and T is the temperature value. Under standard experimental conditions, $I_{sc-STC}$ is the short circuit current, $G_{STC}$ is the irradiance, $T_{STC}$ is the temperature, and α is the temperature coefficient of the short circuit current.

### 7.1. Result of Single Diode (SD) Model

In this model, an RTC France solar cell is used and consists of 26 I–V data pairs. In addition, standard data [70] obtained at 33 °C and 1000 W/m^2^ irradiance are used in this cell model. The best, mean, worst, and standard deviation (std) values obtained by the proposed I-CPA and the basic CPA, SOA, COA, PSO, and DE are given in Table 4. The parameter values obtained by the algorithms according to the best RMSE value are given in Table 5. 

**Figure 7:**
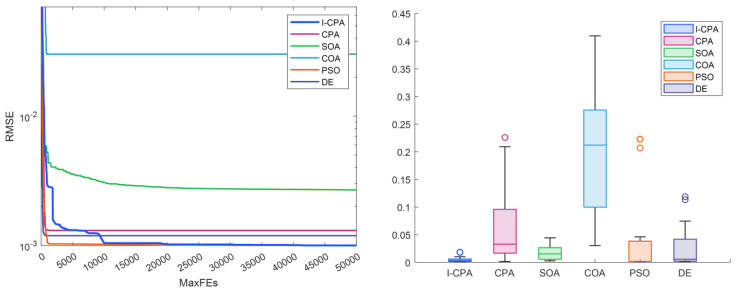
Convergence curves and box plots of algorithms for the SD model.

**Figure 8:**
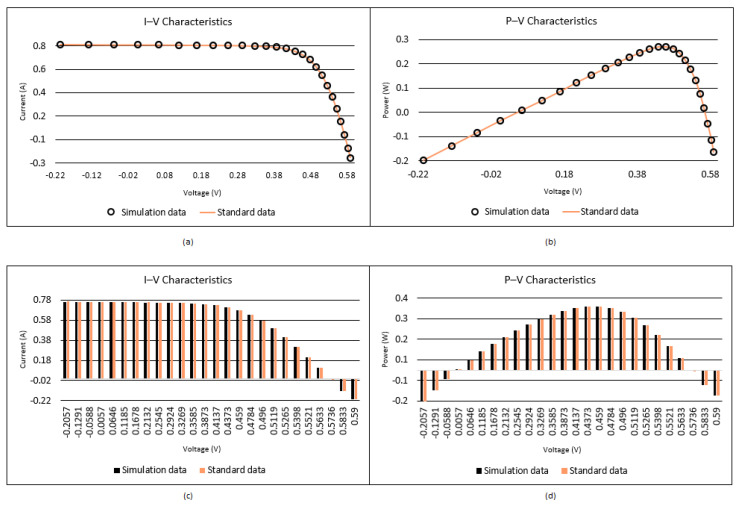
I–V (**a**,**c**) and P–V (**b**,**d**) characteristics of the SD model according to the I-CPA.

When Table 4 and Table 5 are analyzed, it is seen that the best RMSE is obtained by the proposed I-CPA with a value of 9.9861664297 × 10^−4^. Then, the PSO algorithm obtained the second-best RMSE with a value of 1.0013654751 × 10^−3^. Based on the mean RMSE value, it was seen that the I-CPA achieved very good success compared to other algorithms, with a value of 4.4400472660 × 10^−3^. In addition, it was observed that the I-CPA obtained better results compared to other algorithms at std. and worst values. According to the box plots and convergence curve given in Figure 7, it is seen that the I-CPA is more stable than both the basic CPA and the other algorithms. The I–V and P–V characteristic curves obtained with the data generated by the I-CPA are shown in Figure 8. According to the results here, it is seen that the agreement between the simulated data and the standard data is very close.

### 7.2. Result of the Double Diode (DD) Model

In this model, an RTC France solar cell is used and consists of 26 I–V data pairs [70]. The parameter values according to the best RMSE obtained by the proposed I-CPA and the other algorithms are given in Table 6. Also, the best, worst, mean, and std. values produced by the I-CPA and the other algorithms are given in Table 7. The proposed I-CPA obtained a better result than the other algorithms with a value of 1.0252353427 × 10^−3^.

**Figure 9:**
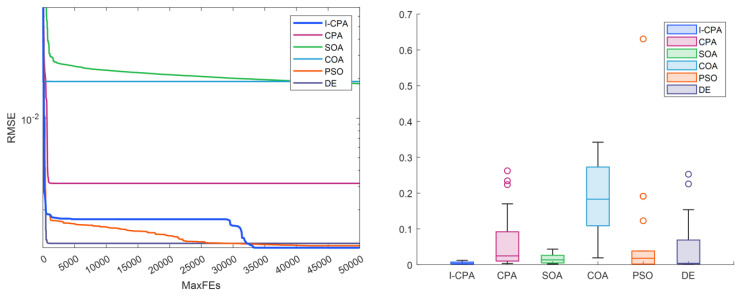
Convergence curves and box plots of algorithms for the DD model.

**Figure 10:**
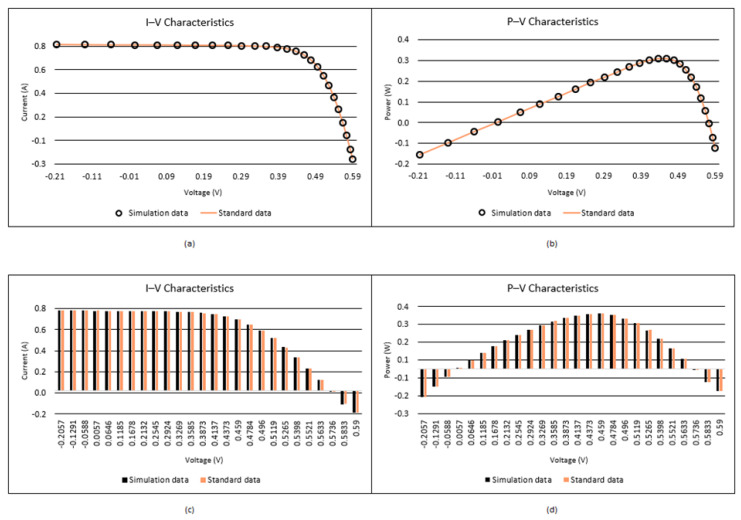
I–V (**a**,**c**) and P–V (**b**,**d**) characteristics according to the I-CPA for the DD model.

The convergence and box plots of the proposed I-CPA and the other algorithms are given in Figure 9. When the box plot is analyzed, it is seen that the I-CPA has a stable structure compared to both the basic CPA and the SOA, COA, PSO, and DE algorithms, while the convergence graphs show that the I-CPA has better convergence performance than other algorithms. The I–V and P–V characteristic curves obtained according to the best value of the I-CPA are given in Figure 10, and it is seen that the standard data and the simulation data obtained by I-CPA coincide with each other.

### 7.3. Result of Photowatt-PWP201 Module (PVM)

In this model, a Photowatt-PWP201 PV module was used and experimental results were obtained using 25 standard data [70] measured at 45 °C and 1000 W/m^2^ irradiance. According to the comparative results given in Table 8, although the results of the proposed I-CPA and DE are close to each other, the I-CPA is successful by producing a better value. Also, when the table is analyzed according to the mean RMSE, it is seen that the I-CPA achieves a better result than the other algorithms. Table 9 shows the parameter values obtained by the algorithms according to the best RMSE value.

**Figure 11:**
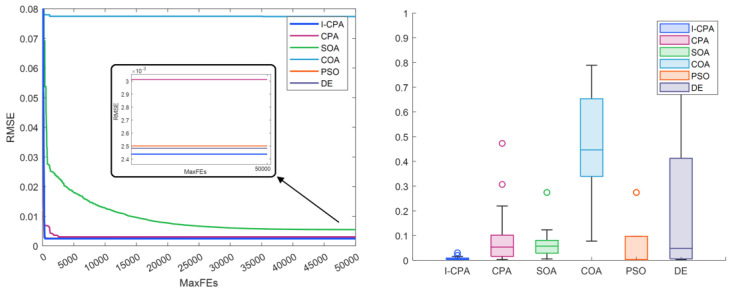
Convergence curves and box plots of algorithms for the PVM.

**Figure 12:**
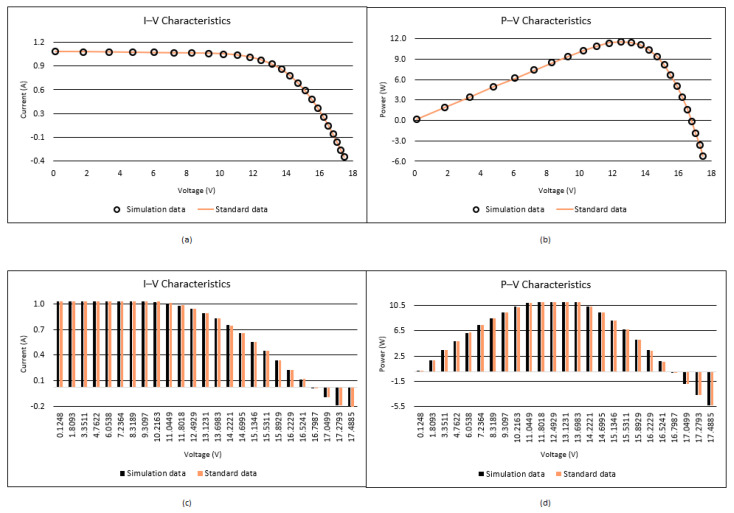
I–V (**a**,**c**) and P–V (**b**,**d**) characteristics according to the I-CPA for the PVM.

The convergence and box plots of the proposed I-CPA and the other algorithms are given in Figure 11, respectively. It is seen from the box plot that the proposed I-CPA is more stable than both the CPA and SOA, COA, PSO, and DE. When the convergence graph is analyzed, it is seen that the COA is stuck to the local minimum after approximately 1000 FEs, and the CPA after approximately 2500 FEs. However, the I-CPA achieved the best result with a faster convergence trend. The I–V and P–V characteristic curves generated according to the data obtained by the I-CPA are given in Figure 12, and it can be said that the simulation data and standard data are compatible.

### 7.4. Result of STM6-40/36 Module

In this section, the STM6-40/36 PV module was used and experimental results were obtained with 20 standard current–voltage data [70] measured at 51 °C and 1000 W/m^2^ irradiance. There are five parameters in this module. The parameter and RMSE values obtained with both the I-CPA and the other algorithms are given in Table 10. In addition, Table 11 shows the best, mean, worst, and std. values obtained by the algorithms. While the proposed I-CPA obtained the best result with a value of 2.1565659255 × 10^-3^, the second-closest value was obtained by the SOA and the algorithm that obtained the third-closest value was the PSO. Based on the mean value, it is seen that the I-CPA is better than other algorithms.

**Figure 13:**
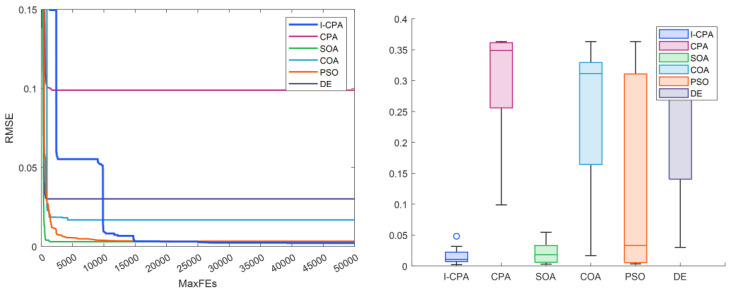
Convergence curves and box plots of algorithms for STM6-40/36.

**Figure 14:**
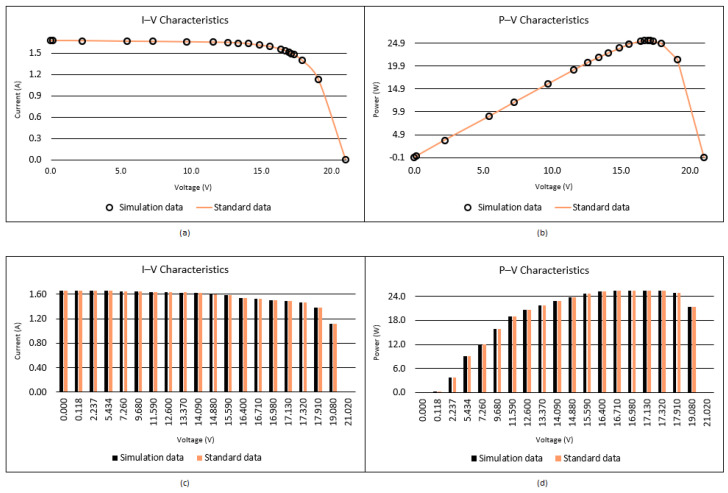
I–V (**a**,**c**) and P–V (**b**,**d**) characteristics according to the I-CPA for STM6-40/36.

Convergence curves obtained according to the best results and box plots showing the stability of the algorithm are given in Figure 13. When the convergence graph is analyzed, it is seen that the CPA, DE and COA get stuck in the local minimum after approximately 1000 FEs, while the I-CPA reaches the best result by continuously escaping from the local minimum. When the box plots are analyzed, it is seen that the I-CPA is more stable than the other algorithms. In Figure 14, the compatibility of the I–V and P–V characteristic curves generated according to the best values of the I-CPA with the standard values is remarkable.

### 7.5. Result of KC200GT Module

In this section, in order to obtain five different parameter values for the KC200GT PV module, analyses were performed using standard data [72] according to different temperature and irradiance values and detailed under two separate titles. In the first section, the temperature value applied to the KC200GT PV module is kept constant at 25 °C and experimental results are obtained according to 200, 400, 600, 800, and 1000 W/m^2^ irradiation values. In the last section, the irradiance value is kept constant at 1000 W/m^2^ and experimental results are obtained according to the temperature values of 25 °C, 50 °C, and 75 °C. The results obtained are presented in the tables and the convergence, box, and I–V, P–V curves are shown in the figures.

#### 7.5.1. Constant Temperature and Different Irradiance Work

The best, mean, std, and worst result values obtained with both the proposed I-CPA and the other algorithms at 25 °C constant temperature and 200, 600, 800, and 1000 W/m^2^ irradiance values are given in Table 12. When the table is analyzed, it is seen that the proposed I-CPA obtained a better result than the other algorithms, with values of 8.7018663730 × 10^−3^ at 200 W/m^2^ irradiance, 2.0547281215 × 10^−2^ at 400 W/m^2^ irradiance, 4.2984541388 × 10^−2^ at 600 W/m^2^ irradiance, 4.6780703134 × 10^−2^ at 800 W/m^2^ irradiance, and 6.8155205337 × 10^−2^ at 1000 W/m^2^ irradiance. At the same time, based on the mean value, it is seen that the I-CPA achieves an even better result. The parameter values obtained by the algorithms according to the best RMSE value are given in Table 13.

**Figure 15:**
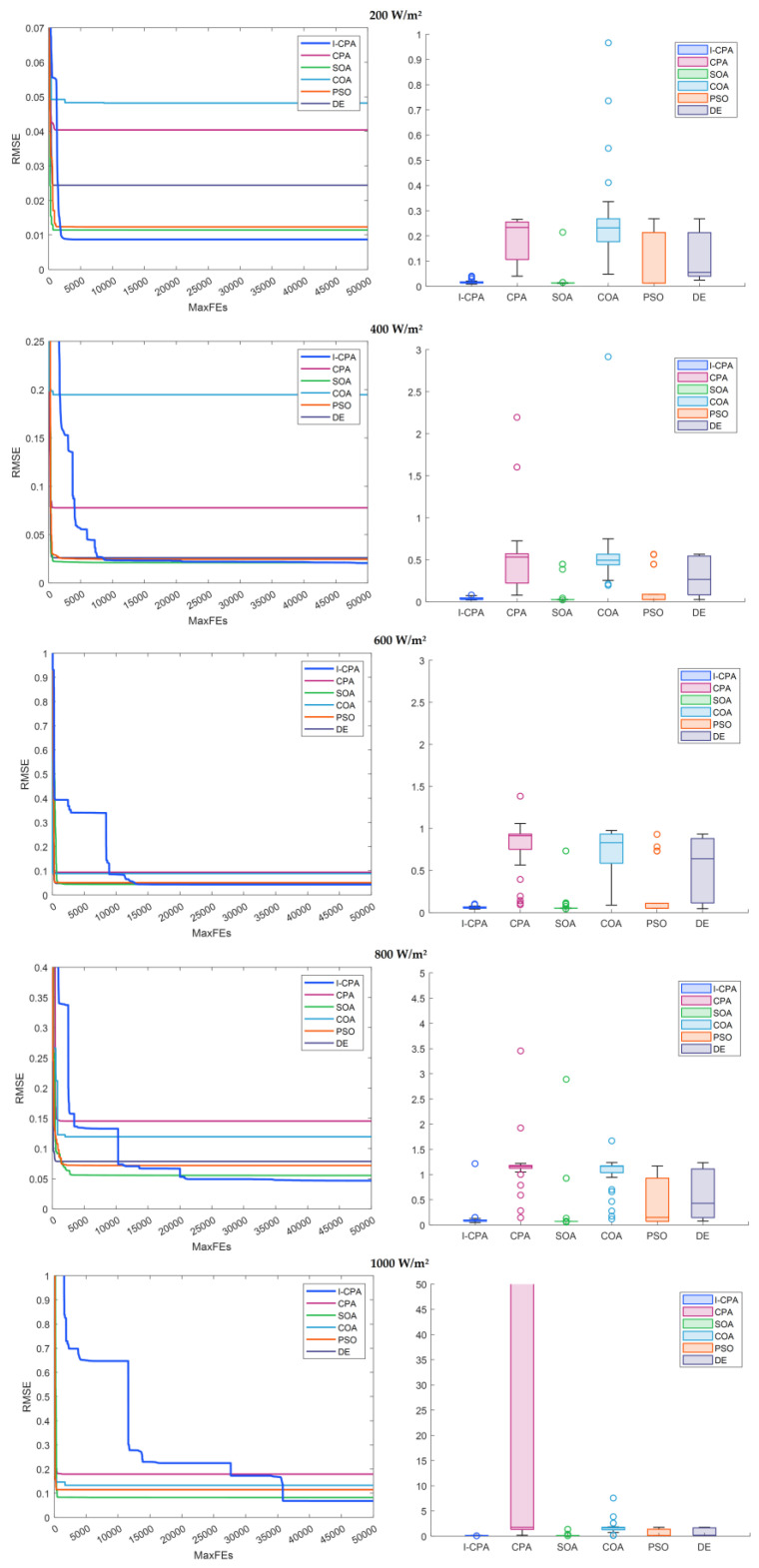
Convergence and box plots of algorithms according to constant temperature and different irradiances.

**Figure 16:**
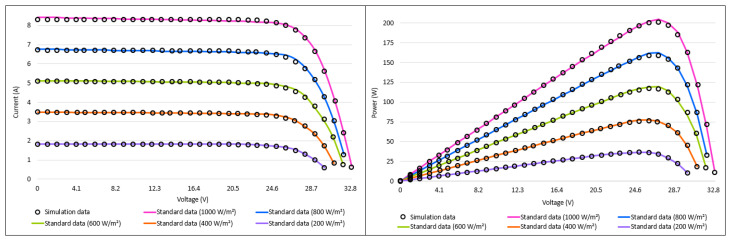
I–V and P–V characteristic curves of the I-CPA according to constant temperature and different irradiances.

The convergence curves obtained according to the best results of the algorithms and the box plots generated according to the results obtained by the algorithms for 30 runtimes are given in Figure 15. When the convergence curves are analyzed, it can be said that the proposed I-CPA converges faster than the other algorithms and tends to avoid local minima. When the box plots are analyzed, it can be said that the proposed I-CPA is more stable than the other algorithms. When the box plots are interpreted for all irradiance values, it is seen that the I-CPA produces more stable results than the basic the CPA. The I–V and P–V characteristic graphs consisting of simulation data obtained by the I-CPA and standard data under constant temperature and different irradiation values are presented in Figure 16. When the figure is analyzed, it is seen that the simulation data of the I-CPA and the standard data are in harmony over the entire voltage range for all cases.

#### 7.5.2. Different Temperature and Constant Irradiance Work

The best, mean, std., and worst result values obtained for 1000 W/m^2^ constant irradiance and different temperature values of 25 °C, 50 °C, and 75 °C are given in Table 14. According to the results in the table, it is seen that the proposed I-CPA produces a better result than the other algorithms with values of 6.8155205337 × 10^−2^ at 25 °C, 6.6338374335 × 10^−2^ at 50 °C, and 6.4612208129 × 10^−2^ at 75 °C. The parameter values produced by the algorithms according to their best result are given in Table 15.

**Figure 17:**
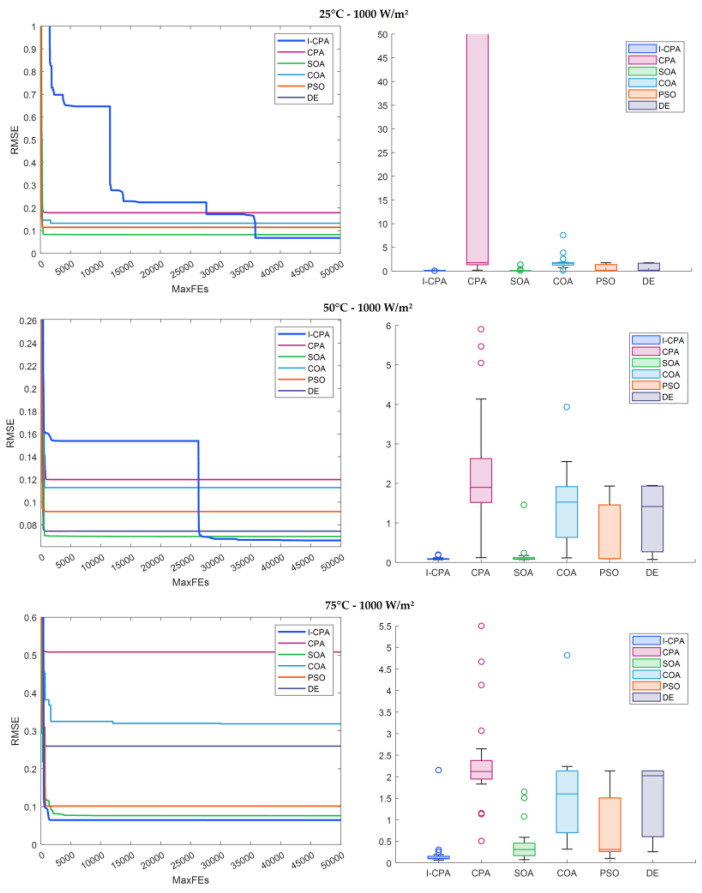
Convergence and box plots of algorithms according to different temperature and constant irradiance.

**Figure 18:**
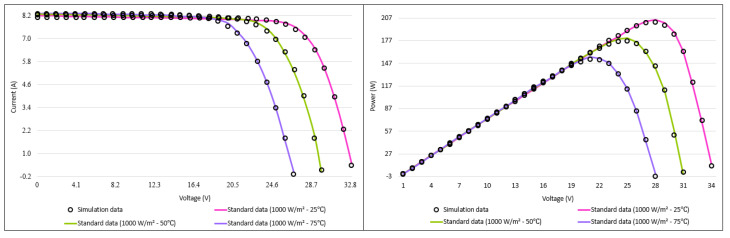
I–V and P–V characteristic curves of the I-CPA according to different temperature and constant irradiance.

When the convergence and box plots given in Figure 17 are analyzed, it can be interpreted from the graph that the I-CPA tends to avoid local minima by showing a better convergence performance than both the basic CPA and the other algorithms. The box plot shows that the I-CPA is more stable than the basic CPA. The I–V and P–V characteristic graphs consisting of simulation data and standard data obtained with the I-CPA under constant irradiance and different temperature values are presented in Figure 18. When the figure is analyzed, it is seen that the simulation data of the I-CPA and the standard data are in harmony over the entire voltage range for all cases.

### 7.6. Statistical Analysis Results of Solar Photovoltaic Modules

The Friedman mean rank test was performed to analyze the performance of the proposed I-CPA and the basic CPA, SOA, COA, PSO, and DE algorithms on solar photovoltaic modules. In the Friedman mean rank test, the algorithm corresponding to the lowest value performs the best. The performance rankings of the algorithms according to the Friedman mean rank statistical test are given in Table 16. In addition, the mean values obtained by the algorithms according to the solar photovoltaic modules are also given in the same table.

When Table 16 is analyzed, the I-CPA is the first algorithm with the best performance in determining the parameter values of solar photovoltaic modules, with an FMR value of 1.09. Then, the SOA is the second algorithm with the best performance with an FMR value of 2.00. The PSO is the third-best-performing algorithm with an FMR value of 3.09. The basic CPA and COA performed similarly to each other, and both algorithms ranked fifth with an FMR value of 5.45. Therefore, the fact that the *p*-value obtained as a result of the Friedman mean rank test is less than 0.05 shows that the I-CPA produces statistically significant results in the parameter identification of solar PV modules compared to other algorithms.

## 8. Conclusions

In this study, the I-CPA method, which is an improved version of the CPA, is proposed to identify the optimum parameter values of solar PV modules. Thus, it aims to both minimize the getting stuck in local minima of the CPA and improve its performance in terms of solution quality in this problem. The performance of the proposed I-CPA method is evaluated on CEC2017 test functions. It is observed that the results obtained with the I-CPA are more successful than the basic CPA. The performance of the I-CPA is also compared with the results of the PSO, DE, SOA, and COA algorithms in the literature. The comparisons show that the proposed I-CPA has a better performance. The Friedman mean rank statistical test was performed to show the ranking of the proposed I-CPA among all algorithms and the significance of the results. As a result of the statistical analyses, the I-CPA ranked first among all algorithms with a score of 1.31 and obtained more successful and statistically significant results. The I-CPA, whose success was tested on CEC2017 functions, was applied to three different models: single diode, double diode, and PV module models. When the results obtained from the models are analyzed, it is seen that the quality of the solutions obtained using the I-CPA method increases compared to the basic CPA. In addition, when the results of the I-CPA are compared with the results of the PSO, DE, SOA, and COA algorithms in the literature, the I-CPA obtained better results. When analyzed in terms of I–V and P–V characteristic curves, it is seen that the simulation data obtained by the I-CPA and the standard data overlap over the entire voltage range. Convergence curves, box plots, and statistical analyses obtained from the experimental results show that the I-CPA produces more significant, stable, and better results than the basic CPA.

In future studies, the I-CPA can be applied to high-dimensional optimization problems and its performance can be measured. In addition, the I-CPA can be hybridized with different optimization algorithms, and performance analyses can be performed on engineering problems. In addition, the I-CPA can be made binary to solve binary optimization problems.

## Figures and Tables

**Table 1:** Unconstrained CEC2017 test functions.

Function	No	Name	*F_min_*
Unimodal	F1	Shifted and Rotated Bent Cigar Function	100
	F3	Shifted and Rotated Zakharov Function	300
Multimodal	F4	Shifted and Rotated Rosenbrock’s Function	400
	F5	Shifted and Rotated Rastrigin’s Functions	500
	F6	Shifted and Rotated Expanded Scaffer’s F6 Function	600
	F7	Shifted and Rotated Lunacek Bi_Rastrigin Function	700
	F8	Shifted and Rotated Non-Continuous Rastrigin’s Function	800
	F9	Shifted and Rotated Levy Function	900
	F10	Shifted and Rotated Schwefel’s Function	1000
Hybrid	F11	Hybrid Function 1 (N = 3)	1100
	F12	Hybrid Function 2 (N = 3)	1200
	F13	Hybrid Function 3 (N = 3)	1300
	F14	Hybrid Function 4 (N = 4)	1400
	F15	Hybrid Function 5 (N = 4)	1500
	F16	Hybrid Function 6 (N = 4)	1600
	F17	Hybrid Function 6 (N = 5)	1700
	F18	Hybrid Function 6 (N = 5)	1800
	F19	Hybrid Function 6 (N = 5)	1900
	F20	Hybrid Function 6 (N = 6)	2000
Composition	F21	Composition Function 1 (N = 3)	2100
	F22	Composition Function 2 (N = 3)	2200
	F23	Composition Function 3 (N = 4)	2300
	F24	Composition Function 4 (N = 4)	2400
	F25	Composition Function 5 (N = 5)	2500
	F26	Composition Function 6 (N = 5)	2600
	F27	Composition Function 7 (N = 6)	2700
	F28	Composition Function 8 (N = 6)	2800
	F29	Composition Function 9 (N = 3)	2900
	F30	Composition Function 10 (N = 3)	3000

Search Range: [−100, 100]^D^, D: 30, MaxFEs: 300,000.

**Table 2:** CEC2017 function results and Friedman mean rank statistics analysis.

No		I-CPA	CPA	SOA	COA	PSO	DE
F1	Mean	**6.58 × 10^9^**	3.55 × 10^10^	4.03 × 10^10^	6.69 × 10^10^	2.25 × 10^10^	3.89 × 10^10^
	Std.	4.51 × 10^9^	1.34 × 10^10^	7.82 × 10^9^	7.40 × 10^9^	1.86 × 10^10^	1.07 × 10^10^
F3	Mean	**5.22 × 10^4^**	1.93 × 10^5^	8.25 × 10^4^	8.69 × 10^4^	9.43 × 10^4^	1.06 × 10^5^
	Std.	8.93 × 10^3^	6.73 × 10^4^	9.09 × 10^3^	7.52 × 10^3^	6.82 × 10^4^	4.30 × 10^4^
F4	Mean	**1.84 × 10^3^**	8.23 × 10^3^	7.86 × 10^3^	1.90 × 10^4^	3.66 × 10^3^	7.65 × 10^3^
	Std.	1.09 × 10^3^	4.29 × 10^3^	2.03 × 10^3^	2.89 × 10^3^	3.45 × 10^3^	2.81 × 10^3^
F5	Mean	**7.28 × 10^2^**	8.47 × 10^2^	7.88 × 10^2^	9.61 × 10^2^	7.36 × 10^2^	7.49 × 10^2^
	Std.	4.90 × 10^1^	6.90 × 10^1^	3.29 × 10^1^	1.99 × 10^1^	5.78 × 10^1^	4.00 × 10^1^
F6	Mean	6.42 × 10^2^	6.62 × 10^2^	6.59 × 10^2^	6.97 × 10^2^	6.41 × 10^2^	**6.40 × 10^2^**
	Std.	7.32 × 10^0^	1.08 × 10^1^	5.42 × 10^0^	5.82 × 10^0^	1.26 × 10^1^	7.83 × 10^0^
F7	Mean	**1.10 × 10^3^**	1.69 × 10^3^	1.26 × 10^3^	1.47 × 10^3^	1.20 × 10^3^	1.46 × 10^3^
	Std.	8.14 × 10^1^	1.68 × 10^2^	6.26 × 10^1^	3.63 × 10^1^	2.36 × 10^2^	1.82 × 10^2^
F8	Mean	**9.93 × 10^2^**	1.09 × 10^3^	1.00 × 10^3^	1.17 × 10^3^	1.02 × 10^3^	1.01 × 10^3^
	Std.	3.51 × 10^1^	5.00 × 10^1^	2.96 × 10^1^	2.33 × 10^1^	5.14 × 10^1^	4.54 × 10^1^
F9	Mean	5.47 × 10^3^	8.06 × 10^3^	**4.74 × 10^3^**	1.18 × 10^4^	6.95 × 10^3^	5.53 × 10^3^
	Std.	1.60 × 10^3^	2.64 × 10^3^	4.85 × 10^2^	8.72 × 10^2^	2.49 × 10^3^	1.51 × 10^3^
F10	Mean	6.00 × 10^3^	7.37 × 10^3^	5.53 × 10^3^	9.28 × 10^3^	**5.35 × 10^3^**	6.14 × 10^3^
	Std.	6.68 × 10^2^	7.43 × 10^2^	6.73 × 10^2^	4.13 × 10^2^	8.02 × 10^2^	7.20 × 10^2^
F11	Mean	**3.49 × 10^3^**	1.84 × 10^4^	5.76 × 10^3^	1.10 × 10^4^	7.03 × 10^3^	1.04 × 10^4^
	Std.	1.81 × 10^3^	7.81 × 10^3^	2.15 × 10^3^	2.36 × 10^3^	1.06 × 10^4^	8.89 × 10^3^
F12	Mean	**6.37 × 10^8^**	6.06 × 10^9^	9.03 × 10^9^	1.96 × 10^10^	2.25 × 10^9^	4.17 × 10^9^
	Std.	7.14 × 10^8^	3.69 × 10^9^	2.89 × 10^9^	3.61 × 10^9^	2.94 × 10^9^	2.17 × 10^9^
F13	Mean	**6.37 × 10^8^**	4.12 × 10^9^	3.92 × 10^9^	1.70 × 10^10^	1.87 × 10^9^	2.47 × 10^9^
	Std.	1.32 × 10^9^	4.33 × 10^9^	3.63 × 10^9^	6.65 × 10^9^	3.92 × 10^9^	3.40 × 10^9^
F14	Mean	**1.02 × 10^6^**	9.13 × 10^6^	1.63 × 10^6^	1.26 × 10^7^	1.19 × 10^6^	1.69 × 10^6^
	Std.	7.34 × 10^5^	1.49 × 10^7^	1.86 × 10^6^	1.69 × 10^7^	2.72 × 10^6^	2.50 × 10^6^
F15	Mean	**7.21 × 10^6^**	6.31 × 10^8^	1.31 × 10^8^	1.70 × 10^9^	1.99 × 10^8^	3.46 × 10^8^
	Std.	1.95 × 10^7^	1.12 × 10^9^	1.86 × 10^8^	1.07 × 10^9^	4.66 × 10^8^	7.19 × 10^8^
F16	Mean	**3.23 × 10^3^**	4.13 × 10^3^	3.65 × 10^3^	7.58 × 10^3^	3.42 × 10^3^	3.34 × 10^3^
	Std.	3.97 × 10^2^	9.84 × 10^2^	5.85 × 10^2^	1.57 × 10^3^	6.41 × 10^2^	5.70 × 10^2^
F17	Mean	**2.31 × 10^3^**	3.01 × 10^3^	3.36 × 10^3^	1.82 × 10^4^	2.70 × 10^3^	2.48 × 10^3^
	Std.	2.16 × 10^2^	6.01 × 10^2^	1.06 × 10^3^	2.03 × 10^4^	3.22 × 10^2^	1.90 × 10^2^
F18	Mean	**4.36 × 10^6^**	4.11 × 10^7^	1.66 × 10^7^	2.32 × 10^8^	1.48 × 10^7^	5.76 × 10^6^
	Std.	5.17 × 10^6^	5.41 × 10^7^	2.31 × 10^7^	2.00 × 10^8^	2.74 × 10^7^	7.42 × 10^6^
F19	Mean	**2.05 × 10^7^**	5.03 × 10^8^	9.01 × 10^7^	1.35 × 10^9^	2.58 × 10^8^	1.79 × 10^8^
	Std.	3.68 × 10^7^	7.12 × 10^8^	1.61 × 10^8^	6.93 × 10^8^	5.33 × 10^8^	3.37 × 10^8^
F20	Mean	**2.65 × 10^3^**	3.02 × 10^3^	2.66 × 10^3^	3.24 × 10^3^	2.67 × 10^3^	2.76 × 10^3^
	Std.	1.59 × 10^2^	2.89 × 10^2^	1.63 × 10^2^	1.83 × 10^2^	2.09 × 10^2^	3.08 × 10^2^
F21	Mean	**2.51 × 10^3^**	2.61 × 10^3^	2.58 × 10^3^	2.82 × 10^3^	2.56 × 10^3^	2.52 × 10^3^
	Std.	3.42 × 10^1^	5.00 × 10^1^	4.91 × 10^1^	6.51 × 10^1^	7.29 × 10^1^	5.00 × 10^1^
F22	Mean	**5.24 × 10^3^**	8.33 × 10^3^	7.63 × 10^3^	1.01 × 10^4^	6.75 × 10^3^	7.15 × 10^3^
	Std.	2.21 × 10^3^	1.35 × 10^3^	6.67 × 10^2^	6.40 × 10^2^	6.71 × 10^2^	8.52 × 10^2^
F23	Mean	**3.08 × 10^3^**	3.27 × 10^3^	3.36 × 10^3^	3.80 × 10^3^	3.13 × 10^3^	3.14 × 10^3^
	Std.	1.22 × 10^2^	1.70 × 10^2^	1.38 × 10^2^	2.49 × 10^2^	1.82 × 10^2^	1.53 × 10^2^
F24	Mean	**3.22 × 10^3^**	3.39 × 10^3^	3.80 × 10^3^	4.05 × 10^3^	3.36 × 10^3^	3.27 × 10^3^
	Std.	8.79 × 10^1^	1.50 × 10^2^	3.66 × 10^2^	2.54 × 10^2^	1.70 × 10^2^	1.51 × 10^2^
F25	Mean	**3.17 × 10^3^**	5.11 × 10^3^	3.95 × 10^3^	5.82 × 10^3^	3.68 × 10^3^	4.38 × 10^3^
	Std.	1.18 × 10^2^	1.01 × 10^3^	3.90 × 10^2^	6.47 × 10^2^	6.60 × 10^2^	6.95 × 10^2^
F26	Mean	**7.52 × 10^3^**	9.14 × 10^3^	9.99 × 10^3^	1.26 × 10^4^	7.97 × 10^3^	8.00 × 10^3^
	Std.	1.06 × 10^3^	1.34 × 10^3^	6.92 × 10^2^	1.20 × 10^3^	1.24 × 10^3^	9.56 × 10^2^
F27	Mean	3.59 × 10^3^	3.83 × 10^3^	4.42 × 10^3^	5.15 × 10^3^	**3.43 × 10^3^**	3.52 × 10^3^
	Std.	1.43 × 10^2^	2.46 × 10^2^	3.32 × 10^2^	6.60 × 10^2^	1.47 × 10^2^	1.28 × 10^2^
F28	Mean	**3.86 × 10^3^**	6.02 × 10^3^	6.18 × 10^3^	8.41 × 10^3^	5.39 × 10^3^	5.80 × 10^3^
	Std.	2.88 × 10^2^	1.23 × 10^3^	8.01 × 10^2^	5.55 × 10^2^	1.75 × 10^3^	1.10 × 10^3^
F29	Mean	4.72 × 10^3^	5.90 × 10^3^	6.08 × 10^3^	1.57 × 10^4^	**4.55 × 10^3^**	4.68 × 10^3^
	Std.	4.08 × 10^2^	8.67 × 10^2^	9.15 × 10^2^	1.21 × 10^4^	4.24 × 10^2^	6.24 × 10^2^
F30	Mean	**4.53 × 10^7^**	4.41 × 10^8^	5.64 × 10^8^	3.19 × 10^9^	2.10 × 10^8^	3.13 × 10^8^
	Std.	8.51 × 10^7^	5.05 × 10^8^	7.74 × 10^8^	1.93 × 10^9^	4.92 × 10^8^	4.36 × 10^8^
FMR		**1.31**	4.72	3.66	5.83	2.45	3.03
Rank		**1**	5	4	6	2	3
*p*-value		**9.87 × 10^-22^**					

The best mean values are marked in bold font. FMR: Friedman mean ranks.

**Table 3:** Boundaries of solar photovoltaic modules.

	Boundaries	$I_{ph\;}\left(A\right)$	$I_{sd,1,2}\;\left(A\right)$	$R_{S}\;\left(Ω\right)$	$R_{sh}\;\left(Ω\right)$	n, $\;n_{1,2}$
RTC France Solar Cell [70]	LB	0	0	0	0	1
	UB	1	1 × 10^−6^	0.5	100	2
Photowatt-PWP201 [70]	LB	0	0	0	0	1
	UB	2	50 × 10^−6^	2	2000	50
STM6-40/36 [70]	LB	0	0	0	0	1
	UB	2	50 × 10^−6^	0.36	1000	60
KC200GT [71]	LB	0	0	0	0	1
	UB	2 × $\;I_{sc}$	100 × 10^−6^	2	5000	5

**Table 4:** RMSE values of the algorithms for the SD model.

Algorithms	Best	Mean	Std.	Worst
I-CPA	**9.9862 × 10^−4^**	**4.4400 × 10^−3^**	4.2832 × 10^−3^	1.8243 × 10^−2^
CPA	1.3053 × 10^−3^	6.8584 × 10^−2^	7.2643 × 10^−2^	2.2585 × 10^−1^
SOA	2.6870 × 10^−3^	1.6503 × 10^−2^	1.2698 × 10^−2^	4.4222 × 10^−2^
COA	3.0192 × 10^−2^	1.9451 × 10^−1^	9.3783 × 10^−2^	4.0959 × 10^−1^
PSO	1.0014 × 10^−3^	4.0018 × 10^−2^	6.1949 × 10^−2^	2.2286 × 10^−1^
DE	1.1893 × 10^−3^	2.3637 × 10^−2^	3.2312 × 10^−2^	1.1858 × 10^−1^

**Table 5:** Parameter values according to the best RMSE for the SD model.

Algorithms	$I_{ph}\;\left(A\right)$	$I_{sd}\;\left(A\right)$	$R_{S}\;\left(Ω\right)$	$R_{sh}\;\left(Ω\right)$	n	RMSE
I-CPA	0.760577	3.2563 × 10^−7^	0.036402	56.80359	1.481923	9.9862 × 10^−4^
CPA	0.759522	3.5336 × 10^−7^	0.036257	79.28121	1.489935	1.3053 × 10^−3^
SOA	0.764164	3.1260 × 10^−7^	0.035694	28.46189	1.478808	2.6870 × 10^−3^
COA	0.728476	8.2413 × 10^−7^	0.017023	35.95890	1.591579	3.0192 × 10^−2^
PSO	0.760735	3.5397 × 10^−7^	0.036011	56.32661	1.490452	1.0014 × 10^−3^
DE	0.760822	4.5474 × 10^−7^	0.034975	63.38279	1.516437	1.1893 × 10^−3^

**Table 6:** Parameter values according to the best RMSE for the DD model.

Algorithms	$I_{ph}\;\left(A\right)$	$I_{sd1}\;\left(A\right)$	$R_{S}\;\left(Ω\right)$	$R_{sh}\;\left(Ω\right)$	$n_{1}$	$I_{sd2}\;\left(A\right)$	$n_{2}$	RMSE
I-CPA	0.760190	4.0418 × 10^−8^	0.036475	63.0435	1.522657	2.8693 × 10^−7^	1.477916	1.0252 × 10^−3^
CPA	0.762026	1.6085 × 10^−7^	0.039435	78.01944	1.760364	1.7933 × 10^−7^	1.427847	3.1788 × 10^−3^
SOA	0.764871	3.6528 × 10^−7^	0.019998	13.00286	1.565339	2.9868 × 10^−7^	1.562413	2.0952 × 10^−3^
COA	0.735841	2.0086 × 10^−7^	0.033445	57.4358	1.857056	2.3340 × 10^−7^	1.456689	1.9054 × 10^−2^
PSO	0.760674	3.9729 × 10^−7^	0.035548	60.2668	1.502309	0.0000 × 10^0^	1.654406	1.0626 × 10^−3^
DE	0.760657	1.5424 × 10^−7^	0.035343	61.3255	1.624442	3.0473 × 10^−7^	1.489441	1.1075 × 10^−3^

**Table 7:** RMSE values of the algorithms for the DD model.

Algorithms	Best	Mean	Std.	Worst
I-CPA	**1.0252 × 10^−3^**	**4.3539 × 10^−3^**	3.0441 × 10^−3^	1.2067 × 10^−2^
CPA	3.1788 × 10^−3^	5.8840 × 10^−2^	7.2786 × 10^−2^	2.6221 × 10^−1^
SOA	2.0952 × 10^−3^	1.5730 × 10^−2^	1.1951 × 10^−2^	4.3416 × 10^−2^
COA	1.9054 × 10^−2^	1.8824 × 10^−1^	9.7086 × 10^−2^	3.4214 × 10^−1^
PSO	1.0626 × 10^−3^	5.7209 × 10^−2^	1.2077 × 10^−1^	6.3074 × 10^−1^
DE	1.1075 × 10^−3^	4.2795 × 10^−2^	6.7470 × 10^−2^	2.5263 × 10^−1^

**Table 8:** RMSE values of the algorithms for the PVM.

Algorithms	Best	Mean	Std.	Worst
I-CPA	**2.4374 × 10^−3^**	**6.7743 × 10^−3^**	6.1563 × 10^−3^	3.0363 × 10^−2^
CPA	3.0119 × 10^−3^	8.9207 × 10^−2^	1.0569 × 10^−1^	4.7271 × 10^−1^
SOA	5.4642 × 10^−3^	8.5049 × 10^−2^	8.8685 × 10^−2^	2.7425 × 10^−1^
COA	7.7377 × 10^−2^	7.0752 × 10^−1^	9.6805 × 10^−1^	4.7377 × 10^0^
PSO	2.5005 × 10^−3^	7.7136 × 10^−2^	1.1219 × 10^−1^	2.7425 × 10^−1^
DE	2.4828 × 10^−3^	3.0656 × 10^−1^	7.3988 × 10^−1^	4.0836 × 10^0^

**Table 9:** Parameter values according to the best RMSE for the PVM.

Algorithms	$I_{ph\;}\left(A\right)$	$I_{sd}\;\left(A\right)$	$R_{S\;}\left(Ω\right)$	$R_{sh}\;\left(Ω\right)$	n	RMSE
I-CPA	1.031157	3.5751 × 10^−6^	1.197135	929.6739	48.74672	2.4374 × 10^−3^
CPA	1.027829	3.8392 × 10^−6^	1.181989	1064.428	49.03548	3.0119 × 10^−3^
SOA	1.047762	2.2434 × 10^−6^	1.210877	304.1560	47.07763	5.4642 × 10^−3^
COA	0.979167	2.1819 × 10^−6^	1.656607	624.8833	47.17717	7.7377 × 10^−2^
PSO	1.029246	4.3374 × 10^−6^	1.178268	1304.384	49.49755	2.5005 × 10^−3^
DE	1.029839	4.1940 × 10^−6^	1.182198	1191.611	49.36549	2.4828 × 10^−3^

**Table 10:** Parameter values according to the best RMSE for the STM6-40/36.

Algorithms	$I_{ph}\;\left(A\right)$	$I_{sd}\;\left(A\right)$	$R_{S}\;\left(Ω\right)$	$R_{sh}\;\left(Ω\right)$	n	RMSE
I-CPA	1.661132	2.6044 × 10^−6^	0.003057	20.60004	1.565799	2.1566 × 10^−3^
CPA	1.564076	2.3197 × 10^−5^	5.63 × 10^−5^	426.2615	1.895378	9.8909 × 10^−2^
SOA	1.662258	4.7862 × 10^−6^	0.000690	22.04669	1.640448	3.0013 × 10^−3^
COA	1.673044	1.8703 × 10^−5^	3.18 × 10^−11^	564.1726	1.834307	1.6812 × 10^−2^
PSO	1.661513	5.5252 × 10^−6^	0.00 × 10^0^	23.71499	1.659002	3.3300 × 10^−3^
DE	1.619320	6.7695 × 10^−6^	1.73 × 10^−5^	999.9999	1.689407	3.0103 × 10^−2^

**Table 11:** RMSE values of the algorithms for the STM6-40/36.

Algorithms	Best	Mean	Std.	Worst
I-CPA	**2.1566 × 10^−3^**	**1.5146 × 10^−2^**	1.0656 × 10^−2^	4.8066 × 10^−2^
CPA	9.8909 × 10^−2^	3.0348 × 10^−1^	8.1796 × 10^−2^	3.6316 × 10^−1^
SOA	3.0013 × 10^−3^	2.0919 × 10^−2^	1.4882 × 10^−2^	5.4448 × 10^−2^
COA	1.6812 × 10^−2^	2.4421 × 10^−1^	1.2490 × 10^−1^	3.6314 × 10^−1^
PSO	3.3300 × 10^−3^	1.3663 × 10^−1^	1.4695 × 10^−1^	3.6317 × 10^−1^
DE	3.0103 × 10^−2^	2.4320 × 10^−1^	1.2111 × 10^−1^	3.6308 × 10^−1^

**Table 12:** RMSE values of the algorithms for constant temperature and different irradiances.

	Algorithms	Best	Mean	Std.	Worst
200 W/m^2^					
	I-CPA	**8.7019 × 10^−3^**	**1.7585 × 10^−2^**	7.6136 × 10^−3^	4.0644 × 10^−2^
	CPA	4.0412 × 10^−2^	1.8935 × 10^−1^	7.6538 × 10^−2^	2.6581 × 10^−1^
	SOA	1.1437 × 10^−2^	1.9814 × 10^−2^	3.6146 × 10^−2^	2.1440 × 10^−1^
	COA	4.8206 × 10^−2^	2.9368 × 10^−1^	2.3204 × 10^−1^	1.0333 × 10^0^
	PSO	1.2346 × 10^−2^	9.2294 × 10^−2^	1.0118 × 10^−1^	2.6786 × 10^−1^
	DE	2.4422 × 10^−2^	1.2177 × 10^−1^	9.1937 × 10^−2^	2.6774 × 10^−1^
400 W/m^2^					
	I-CPA	**2.0547 × 10^−2^**	**3.7445 × 10^−2^**	1.5605 × 10^−2^	8.0397 × 10^−2^
	CPA	7.7800 × 10^−2^	5.3220 × 10^−1^	4.1892 × 10^−1^	2.1953 × 10^0^
	SOA	2.0999 × 10^−2^	9.3643 × 10^−2^	1.5289 × 10^−1^	4.4701 × 10^−1^
	COA	1.9475 × 10^−1^	5.5592 × 10^−1^	4.5848 × 10^−1^	2.9165 × 10^0^
	PSO	2.4469 × 10^−2^	1.1976 × 10^−1^	1.7069 × 10^−1^	5.6505 × 10^−1^
	DE	2.6128 × 10^−2^	3.1305 × 10^−1^	2.2140 × 10^−1^	5.6507 × 10^−1^
600 W/m^2^					
	I-CPA	**4.2985 × 10^−2^**	**6.3347 × 10^−2^**	1.6160 × 10^−2^	1.0384 × 10^−1^
	CPA	9.3935 × 10^−2^	1.0959 × 10^2^	5.8594 × 10^2^	3.2650 × 10^3^
	SOA	4.3828 × 10^−2^	1.0276 × 10^−1^	1.6895 × 10^−1^	7.3125 × 10^−1^
	COA	8.8716 × 10^−2^	1.1665 × 10^0^	1.2086 × 10^0^	4.5504 × 10^0^
	PSO	5.1102 × 10^−2^	3.3265 × 10^−1^	8.2807 × 10^−1^	4.5504 × 10^0^
	DE	4.7580 × 10^−2^	5.7519 × 10^−1^	4.8638 × 10^−1^	2.3933 × 10^0^
800 W/m^2^					
	I-CPA	**4.6781 × 10^−2^**	**1.2417 × 10^−1^**	2.0414 × 10^−1^	1.2164 × 10^0^
	CPA	1.4547 × 10^−1^	2.6183 × 10^15^	1.4099 × 10^16^	7.8541 × 10^16^
	SOA	5.5369 × 10^−2^	2.5267 × 10^−1^	5.5278 × 10^−1^	2.8901 × 10^0^
	COA	1.1941 × 10^−1^	1.3369 × 10^0^	1.3180 × 10^0^	6.1055 × 10^0^
	PSO	7.1854 × 10^−2^	4.5482 × 10^−1^	4.2569 × 10^−1^	1.1703 × 10^0^
	DE	7.8629 × 10^−2^	6.0898 × 10^−1^	4.6163 × 10^−1^	1.2369 × 10^0^
1000 W/m^2^					
	I-CPA	**6.8155 × 10^−2^**	**1.2635 × 10^−1^**	2.1128 × 10^−2^	1.6568 × 10^−1^
	CPA	1.7914 × 10^−1^	2.4882 × 10^34^	1.3396 × 10^35^	7.4627 × 10^35^
	SOA	8.2649 × 10^−2^	2.4378 × 10^−1^	3.7978 × 10^−1^	1.3716 × 10^0^
	COA	1.3297 × 10^−1^	1.7254 × 10^0^	1.2918 × 10^0^	7.5683 × 10^0^
	PSO	1.1458 × 10^−1^	5.8412 × 10^−1^	6.9455 × 10^−1^	1.7602 × 10^0^
	DE	1.1517 × 10^−1^	6.8757 × 10^−1^	6.9629 × 10^−1^	1.7615 × 10^0^

**Table 13:** Parameter values of the PV module according to the best RMSE of the algorithms under constant temperature and different irradiance conditions.

Algorithms		$I_{ph\;}\left(A\right)$	$I_{sd\;}\left(A\right)$	$R_{S}\;\left(Ω\right)$	$R_{sh}\;\left(Ω\right)$	n	RMSE
200 W/m^2^							
	I-CPA	1.577919	1.8993 × 10^−7^	0.009315	5000.00	1.390763	8.7019 × 10^−3^
	CPA	1.599835	5.1585 × 10^−5^	9.32 × 10^−6^	2470.12	2.164018	4.0412 × 10^−2^
	SOA	1.579777	1.1965 × 10^−6^	0.002324	1221.57	1.567724	1.1437 × 10^−2^
	COA	1.625524	4.9398 × 10^−5^	0.000841	2466.46	2.141879	4.8206 × 10^−2^
	PSO	1.579721	1.8262 × 10^−6^	0.00 × 10^0^	5000.00	1.614168	1.2346 × 10^−2^
	DE	1.589322	1.2742 × 10^−5^	0.00 × 10^0^	3024.83	1.893861	2.4422 × 10^−2^
400 W/m^2^							
	I-CPA	3.253889	3.2070 × 10^−7^	0.00324	6.598593	1.413711	2.0547 × 10^−2^
	CPA	3.247364	3.7822 × 10^−5^	0.00166	4390.309	2.025899	7.7800 × 10^−2^
	SOA	3.228092	8.5527 × 10^−7^	0.00209	17.78862	1.503491	2.0999 × 10^−2^
	COA	3.111251	3.0476 × 10^−5^	0.00693	1436.038	2.053825	1.9475 × 10^−1^
	PSO	3.229227	2.0840 × 10^−6^	0.00 × 10^0^	16.56421	1.594609	2.4469 × 10^−2^
	DE	3.220904	3.7452 × 10^−6^	9.53 × 10^−6^	2294.147	1.663803	2.6128 × 10^−2^
600 W/m^2^							
	I-CPA	4.856086	1.1361 × 10^−6^	0.001913	10.81073	1.526247	4.2985 × 10^−2^
	CPA	4.900939	6.0026 × 10^−5^	1.82 × 10^−6^	3911.121	2.069565	9.3935 × 10^−2^
	SOA	4.848421	2.4426 × 10^−6^	0.001801	48.28901	1.607485	4.3828 × 10^−2^
	COA	4.843326	4.1875 × 10^−5^	9.73 × 10^−5^	1789.595	2.005826	8.8716 × 10^−2^
	PSO	4.848333	7.6747 × 10^−6^	0.00 × 10^0^	75.67750	1.741967	5.1102 × 10^−2^
	DE	4.850289	4.8496 × 10^−6^	1.10 × 10^−3^	3944.496	1.686342	4.7580 × 10^−2^
800 W/m^2^							
	I-CPA	6.481358	1.4688 × 10^−7^	0.003116	9.06509	1.335718	4.6781 × 10^−2^
	CPA	6.533486	7.9757 × 10^−5^	0.000185	2506.688	2.088066	1.4547 × 10^−1^
	SOA	6.487515	6.4209 × 10^−7^	0.002180	10.29268	1.456419	5.5369 × 10^−2^
	COA	6.523348	4.5172 × 10^−5^	2.46 × 10^−20^	2483.004	1.977255	1.1941 × 10^−1^
	PSO	6.485372	5.5691 × 10^−6^	0.00 × 10^0^	14.02724	1.676213	7.1854 × 10^−2^
	DE	6.484879	1.3011 × 10^−5^	0.00 × 10^0^	4443.546	1.787232	7.8629 × 10^−2^
1000 W/m^2^							
	I-CPA	8.061536	7.5512 × 10^−8^	0.003449	4995.431	1.289751	6.8155 × 10^−2^
	CPA	8.121738	1.9898 × 10^−5^	0.001601	546.2093	1.856372	1.7914 × 10^−1^
	SOA	8.117739	1.0818 × 10^−6^	0.002224	15.27229	1.505406	8.2649 × 10^−2^
	COA	8.167953	4.9748 × 10^−5^	0.00 × 10^0^	2487.763	1.982407	1.3297 × 10^−1^
	PSO	8.114500	1.9540 × 10^−5^	0.00 × 10^0^	5000.00	1.837472	1.1458 × 10^−1^
	DE	8.119760	2.3241 × 10^−5^	0.00 × 10^0^	2537.75	1.862979	1.1517 × 10^−1^

**Table 14:** RMSE values of the algorithms for different temperature and constant irradiance.

	Algorithms	Best	Mean	Std.	Worst
25 °C—1000 W/m^2^					
	I-CPA	**6.8155 × 10^−2^**	**1.2635 × 10^−1^**	2.1128 × 10^−2^	1.6568 × 10^−1^
	CPA	1.7914 × 10^−1^	2.4882 × 10^34^	1.3396 × 10^35^	7.4627 × 10^35^
	SOA	8.2649 × 10^−2^	2.4378 × 10^−1^	3.7978 × 10^−1^	1.3716 × 10^0^
	COA	1.3297 × 10^−1^	1.7254 × 10^0^	1.2918 × 10^0^	7.5683 × 10^0^
	PSO	1.1458 × 10^−1^	5.8412 × 10^−1^	6.9455 × 10^−1^	1.7602 × 10^0^
	DE	1.1517 × 10^−1^	6.8757 × 10^−1^	6.9629 × 10^−1^	1.7615 × 10^0^
50 °C—1000 W/m^2^					
	I-CPA	**6.6338 × 10^−2^**	**9.3289 × 10^−2^**	2.7757 × 10^−2^	1.9511 × 10^−1^
	CPA	1.1992 × 10^−1^	8.0406 × 10^30^	4.3300 × 10^31^	2.4122 × 10^32^
	SOA	6.9765 × 10^−2^	1.8893 × 10^−1^	3.4039 × 10^−1^	1.4563 × 10^0^
	COA	1.1288 × 10^−1^	1.3781 × 10^0^	8.1909 × 10^−1^	3.9345 × 10^0^
	PSO	9.1813 × 10^−2^	9.1974 × 10^−1^	1.4235 × 10^0^	7.5736 × 10^0^
	DE	7.4535 × 10^−2^	1.2903 × 10^0^	1.2307 × 10^0^	6.4153 × 10^0^
75 °C—1000 W/m^2^					
	I-CPA	**6.4612 × 10^−2^**	**1.9626 × 10^−1^**	3.6800 × 10^−1^	2.1540 × 10^0^
	CPA	5.0825 × 10^−1^	2.1560 × 10^4^	1.1609 × 10^5^	6.4674 × 10^5^
	SOA	7.6140 × 10^−2^	4.3558 × 10^−1^	4.2661 × 10^−1^	1.6534 × 10^0^
	COA	3.1861 × 10^−1^	1.5048 × 10^0^	9.3394 × 10^−1^	4.8212 × 10^0^
	PSO	1.0167 × 10^−1^	7.4441 × 10^−1^	6.3584 × 10^−1^	2.1347 × 10^0^
	DE	2.5991 × 10^−1^	1.4281 × 10^0^	7.9062 × 10^−1^	2.1360 × 10^0^

**Table 15:** Parameter values of the PV module according to the best RMSE of the algorithms under different temperature and constant irradiance conditions.

Algorithms		$I_{ph}\;\left(A\right)$	$I_{sd}\;\left(A\right)$	$R_{S}\;\left(Ω\right)$	$R_{sh}\;\left(Ω\right)$	n	RMSE
25 °C—1000 W/m^2^							
	I-CPA	8.061536	7.5512 × 10^−8^	0.003449	4995.431	1.289751	6.8155 × 10^−2^
	CPA	8.121738	1.9898 × 10^−5^	0.001601	546.2093	1.856372	1.7914 × 10^−1^
	SOA	8.1177390	1.0818 × 10^−6^	0.002224	15.27229	1.505406	8.2649 × 10^−2^
	COA	8.1679533	4.9748 × 10^−5^	0.00 × 10^0^	2487.763	1.982407	1.3297 × 10^−1^
	PSO	8.1145002	1.9540 × 10^−5^	0.00 × 10^0^	5000.000	1.837472	1.1458 × 10^−1^
	DE	8.1197601	2.3241 × 10^−5^	0.00 × 10^0^	2537.752	1.862979	1.1517 × 10^−1^
50 °C—1000 W/m^2^							
	I-CPA	8.217318	4.6475 × 10^−6^	0.002365	8.286305	1.379142	6.6338 × 10^−2^
	CPA	8.317071	5.3480 × 10^−5^	0.001114	3953.827	1.657883	1.1992 × 10^−1^
	SOA	8.213238	9.6663 × 10^−6^	0.001949	12.73250	1.574289	6.9765 × 10^−2^
	COA	8.162757	4.9712 × 10^−5^	1.41 × 10^−8^	2485.616	1.646706	1.1288 × 10^−1^
	PSO	8.223759	1.0000 × 10^−4^	2.33 × 10^−4^	5000.000	1.749940	9.1813 × 10^−2^
	DE	8.203875	2.1073 × 10^−5^	1.49 × 10^−3^	5000.000	1.539649	7.4535 × 10^−2^
75 °C—1000 W/m^2^							
	I-CPA	8.274836	7.2027 × 10^−6^	0.004698	38.39862	1.187854	6.4612 × 10^−2^
	CPA	8.265985	8.1748 × 10^−5^	0.005869	3865.020	1.476085	5.0825 × 10^−1^
	SOA	8.285779	1.8963 × 10^−5^	0.004161	55.04236	1.489774	7.6140 × 10^−2^
	COA	8.225573	1.0000 × 10^−4^	1.77 × 10^−21^	5000.000	1.449672	3.1861 × 10^−1^
	PSO	8.304616	1.0000 × 10^−4^	3.02 × 10^−3^	5000.000	1.461759	1.0167 × 10^−1^
	DE	8.189925	6.7452 × 10^−5^	9.52 × 10^−4^	2751.274	1.405813	2.5991 × 10^−1^

**Table 16:** Mean values of solar photovoltaic modules and Friedman mean rank statistics results.

Modules	I-CPA	CPA	SOA	COA	PSO	DE
SD	4.44 × 10^−3^	6.86 × 10^−2^	1.65 × 10^−2^	1.95 × 10^−1^	4.00 × 10^−2^	2.36 × 10^−2^
DD	4.35 × 10^−3^	5.88 × 10^−2^	1.57 × 10^−2^	1.88 × 10^−1^	5.72 × 10^−2^	4.28 × 10^−2^
PVM	6.77 × 10^−3^	8.92 × 10^−2^	8.50 × 10^−2^	7.08 × 10^−1^	7.71 × 10^−2^	3.07 × 10^−1^
STM6-40/36	1.51 × 10^−2^	3.03 × 10^−1^	2.09 × 10^−2^	2.44 × 10^−1^	1.37 × 10^−1^	2.43 × 10^−1^
KC200GT-200 W/m^2^-25 °C	1.76 × 10^−2^	1.89 × 10^−1^	1.98 × 10^−2^	2.94 × 10^−1^	9.23 × 10^−2^	1.22 × 10^−1^
KC200GT-400 W/m^2^-25 °C	3.74 × 10^−2^	5.32 × 10^−1^	9.36 × 10^−2^	5.56 × 10^−1^	1.20 × 10^−1^	3.13 × 10^−1^
KC200GT-600 W/m^2^-25 °C	6.33 × 10^−2^	1.10 × 10^2^	1.03 × 10^−1^	1.17 × 10^0^	3.33 × 10^−1^	5.75 × 10^−1^
KC200GT-800 W/m^2^-25 °C	1.24 × 10^−1^	2.62 × 10^15^	2.53 × 10^−1^	1.34 × 10^0^	4.55 × 10^−1^	6.09 × 10^−1^
KC200GT-1000 W/m^2^-25 °C	1.26 × 10^−1^	2.49 × 10^34^	2.44 × 10^−1^	1.73 × 10^0^	5.84 × 10^−1^	6.88 × 10^−1^
KC200GT-1000 W/m^2^-50 °C	9.33 × 10^−2^	8.04 × 10^30^	1.89 × 10^−1^	1.38 × 10^0^	9.20 × 10^−1^	1.29 × 10^0^
KC200GT-1000 W/m^2^-75 °C	1.96 × 10^−1^	2.16 × 10^4^	4.36 × 10^−1^	1.50 × 10^0^	6.36 × 10^−1^	1.43 × 10^0^
FMR	**1.09**	5.45	2.00	5.45	3.09	3.91
Rank	**1**	5	2	5	3	4
*p*-value	**8.86 × 10^−10^**					

FMR: Friedman mean ranks.

## Data Availability

No new data were created or analyzed in this study. Data sharing is not applicable to this article.
